# Olaparib and somatic BRCA mutations

**DOI:** 10.18632/oncotarget.18419

**Published:** 2017-06-08

**Authors:** Angela George, Susana Banerjee, Stan Kaye

**Affiliations:** Gynaecology Unit, Royal Marsden NHS Foundation Trust, Sutton, UK

**Keywords:** olaparib, ovarian, somatic, BRCA, mutations

Ten years after the publication of the seminal laboratory studies, which demonstrated the exquisite sensitivity of *BRCA* mutated cells to poly (ADP ribose) polymerase (PARP) inhibition, the first PARP inhibitor, olaparib received regulatory approval for the maintenance therapy of relapsed platinum-sensitive *BRCA* mutated ovarian cancer. Approval was gained for patients with both germline and somatic mutations in *BRCA1* or *BRCA2,* based on the results of Study 19, which randomised 265 patients with relapsed disease to either olaparib maintenance treatment (400mg bd capsules) or placebo. A pre-planned retrospective analysis on 254 of these indicated that 136 (55%) of patients had a *BRCA1* or *BRCA2* mutation, including 18 (7%) with a somatic (tumour) mutation without a reported germ-line mutation. The extent of benefit for olaparib in *BRCA* mutated cases as measured by the primary endpoint (progression-free survival) was remarkable with a hazard ratio (HR) of 0.18 and median progression-free survival (median PFS) increasing from 4.3 to 11.2 months [1]. Although the number of cases was small, a subgroup analysis in those with somatic not germline *BRCA* mutations indicated a comparable benefit [2].

The current paper by Dougherty et al, examines this cohort in further detail and uses a new computational algorithm to determine the source of mutation in patients without matched normal samples, to identify somatic versus germline mutations- the somatic germ-line zygosity (SGZ) algorithm. In doing so, they were able to identify two further cases of somatic mutations, with sub-clonal *BRCA2* mutations. None of the known germline mutations was predicted to be somatic using the algorithm, and this therefore provides a possible avenue for future testing of patients without matched samples. Further information on this (as yet unpublished) algorithm will be of great interest in those wishing to perform retrospective analysis on such patients. Their analysis confirms the high level of efficacy for olaparib in this subgroup of 20, with a HR of 0.23 in respect of the PFS comparison [3].

How do these results compare with those using other PARP inhibitors – niraparib and rucaparib? For niraparib, a similar randomised maintenance study in platinum-sensitive ovarian cancer (NOVA) of 553 patients, included 47 with somatic *BRCA* mutations; in these the HR for benefit was 0.27 with median PFS increasing from 11 to 20.9 months [4]. For rucaparib, data are available for response in patients with platinum-sensitive measurable advanced disease; for 19 patients with somatic *BRCA* mutation the RECIST response rate was 74% which was similar to those with germ line *BRCA* mutations (85%) [5].

There seems little doubt that the remarkable efficacy of PARP inhibitors in patients with ovarian cancer will extend beyond those with germline *BRCA* mutations, to include others with characteristics of homologous recombination deficiency (HRD) including those with somatic *BRCA* mutations. For every 3-4 germline *BRCA* mutated patients there is probably an additional one with a somatic mutation, and this represents a significant number of cases who have the potential to benefit most from PARP inhibitors. Further information will be needed from on-going trials in order to determine whether the duration of benefit in patients with somatic and germline mutations is similar. The question also arises as to when patients should be tested for somatic mutations – i.e. will the use of archival tissue risk missing those patients who acquire somatic mutations later in their disease pathway? Dougherty et al point out that the clonality and high biallelic inactivation frequency observed suggests that somatic *BRCA* loss is an early event. This suggests that there is limited use in retesting for somatic *BRCA* mutations with fresh biopsies at each relapse, although the data from ARIEL 2 [5]. indicate that the debate on this issue is likely to continue. It also seems likely that the extent of tumour heterogeneity, which is increasingly recognised as a key factor in determining the ultimate efficacy of PARP inhibition, will relate to whether somatic *BRCA* loss is an early or late event.

A minority of women with ovarian cancer have now received this therapy for several years and remain in remission – understanding the tumour characteristics in these ‘super-responder’ cases, including the frequency of somatic as well as germ-line *BRCA* mutations will be of special interest [6]. Such information may also give further insights into mechanisms of resistance, and ways in which this could be overcome in future.

A key current issue is whether cancers with other mechanisms causing HRD can be identified and selected for PARP inhibition. For both niraparib and rucaparib, an HRD assay incorporating measures of loss of heterozygosity has been utilised with mixed results. Of note, there are multiple different HRD assays that have been developed and utilised. However, there is some lack of clarity in the methods used to assess HRD, making it difficult to make direct comparisons and raising the possibility that patients could be deemed HRD deficient by one assay, yet HRD proficient by others. In other diseases, i.e. prostate cancer, a genomic (NGS) assay has identified a molecular fingerprint with promising predictive potential for PARP inhibitor sensitivity [7]. In essence this is now the major target, i.e. how to extend the population of patients beyond those with both germ-line and somatic *BRCA* mutations, to more cases who could benefit from PARP inhibition. Meantime it seems clear that somatic, as well as germ line *BRCA* mutation analysis, should become standard of care for the management of women with ovarian cancer [8].
